# Corrigendum to: Cohort profile: Extended Cohort for E-health, Environment and DNA (EXCEED)

**DOI:** 10.1093/ije/dyz175

**Published:** 2019-07-31

**Authors:** Catherine John, Nicola F Reeve, Robert C Free, Alexander T Williams, Ioanna Ntalla, Aliki-Eleni Farmaki, Jane Bethea, Linda M Barton, Nick Shrine, Chiara Batini, Richard Packer, Sarah Terry, Beverley Hargadon, Qingning Wang, Carl A Melbourne, Emma L Adams, Catherine E Bee, Kyla Harrington, José Miola, Nigel J Brunskill, Christopher E Brightling, Julian Barwell, Susan E Wallace, Ron Hsu, David J Shepherd, Edward J Hollox, Louise V Wain, Martin D Tobin

**Affiliations:** 1 Department of Health Sciences, University of Leicester, Leicester, UK; 2 NIHR Leicester Biomedical Research Centre, University of Leicester, Leicester, UK; 3 Department of Respiratory Sciences, University of Leicester, Leicester, UK; 4 Department of Clinical Pharmacology, William Harvey Research Institute, Barts & The London Medical School, Queen Mary University of London, Charterhouse Square, London, UK; 5 Department of Population Science and Experimental Medicine, Institute of Cardiovascular Science, University College London, London, UK; 6 Department of Haematology, University Hospitals of Leicester NHS Trust, Leicester, UK; 7 Leicester Law School, University of Leicester, Leicester, UK; 8 Department of Cardiovascular Sciences University of Leicester, Leicester, UK; 9 Department of Genetics and Genome Biology, University of Leicester, Leicester, UK

First published online: 7 May 2019, *Int J Epidemiol* 2019; 48: 678–79j. doi: https://doi.org/10.1093/ije/dyz073

An author of this cohort profile, Ioanna Ntalla, was unintentionally omitted from the author list. Dr Ntalla made a substantial contribution to the design and implementation of the study and meets the criteria for authorship.

The author list has been corrected.

